# Just the Facts: Airway management during the coronavirus disease 2019 (COVID-19) pandemic

**DOI:** 10.1017/cem.2020.353

**Published:** 2020-03-30

**Authors:** George Kovacs, Nicholas Sowers, Samuel Campbell, James French, Paul Atkinson

**Affiliations:** * Department of Emergency Medicine, Dalhousie University, Halifax, NS; † Department of Emergency Medicine, Dalhousie University, Saint John Regional Hospital, NB

**Keywords:** Airway management, coronavirus, hazard control measures

## Abstract

A previously healthy 42-year-old male developed a fever and cough shortly after returning to Canada from overseas. Initially, he had mild upper respiratory tract infection symptoms and a cough. He was aware of the coronavirus disease-2019 (COVID-19) and the advisory to self-isolate and did so; however, he developed increasing respiratory distress over several days and called 911. On arrival at the emergency department (ED), his heart rate was 130 beats/min, respiratory rate 32 per/min, and oxygenation saturation 82% on room air. As per emergency medical services (EMS) protocol, they placed him on nasal prongs under a surgical mask at 5 L/min and his oxygen saturation improved to 86%.

## CASE

A previously healthy 42-year-old male developed a fever and cough shortly after returning to Canada from overseas. Initially, he had mild upper respiratory tract infection symptoms and a cough. He was aware of the coronavirus disease-2019 (COVID-19) and the advisory to self-isolate and did so; however, he developed increasing respiratory distress over several days and called 911. On arrival at the emergency department (ED), his heart rate was 130 beats/min, respiratory rate 32 per/min, and oxygenation saturation 82% on room air. As per emergency medical services (EMS) protocol, they placed him on nasal prongs under a surgical mask at 5 L/min and his oxygen saturation improved to 86%.

## KEY CLINICAL QUESTIONS

1.: **What COVID-19 patients should be considered for intubation?**

*Answer:* While a majority of patients will have minor illnesses and never present to the ED, the progression of disease for those who may ultimately require intensive care unit level of care is relatively slow (9–10 days).^1^ However, patients may deteriorate during self-isolation and therefore present relatively late, in acute distress. Reports from areas with high incidence of COVID-19 infection inform us that patients not uncommonly present with impressively low saturations on supplemental oxygen, and, while they are symptomatic with dyspnea, they are not necessarily “altered”^2^^,^^3^ (personal communications, Italy). Careful escalation with oxygen therapy and other resuscitation measures should continue.^4^ Delays in making the decision to intubate must be balanced against the risk of later managing a crashing patient in an uncontrolled scenario.^3^ COVID-19 pneumonia patients in respiratory distress with persistent hypoxemia and who are showing signs of fatigue (altered mental status) despite escalation of oxygen therapy (i.e., non-rebreather face mask at 15 L/min) are at significant risk for requiring urgent intubation.

2.: **What's different about intubating patients who may have COVID-19?**

**Figure 1.**
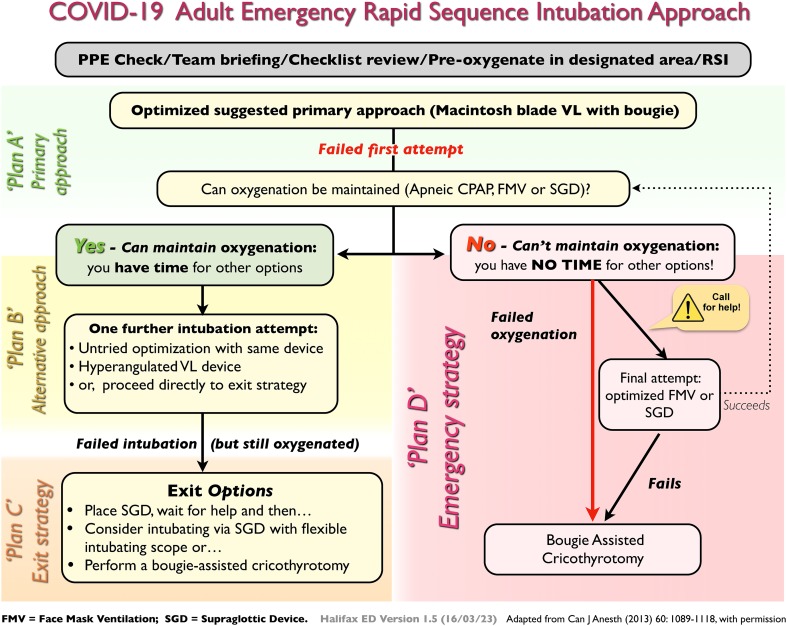
Emergency RSI airway management visual aid.

*Answer:* Simply put, it's the *same* for the most part with a few important differences. We're performing a rapid sequence intubation (RSI) with the goal of a high first-pass success (FPS) rate with your “team” that you are familiar with. The accompanying algorithm is very similar in approach to what most emergency medicine physicians do currently (Figure 1). It's *different* in that airway management of COVID-19 patients requires a paradigm shift from a focus primarily on patient-oriented outcomes to one that focuses on provider safety. Caregivers of COVID-19 patients are at increased risk of contracting the virus primarily by contact/droplet spread. Airway management additionally poses an increased risk to the provider for two major reasons: 1) These sick patients likely carry a greater viral load and 2) conventionally performed airway procedures will produce airborne particles (aerosol generating procedures [AGPs]).^5^ Another major reason why airway management in COVID-19 patients is different relates to the details and sequencing related to provider safety. It's the small stuff, such as paying attention, having lean but complete equipment, knowing how to manage oxygen flow safely, and routinely using a checklist. Lastly, COVID-19 airway management is different because we are forced by circumstance to commit to processes and procedures using evidence that is at best, Level C (low quality, consensus documents expert opinion).

3.: **How do I protect myself and my team?**

*Answer:* There is considerable discussion and concern amongst healthcare providers around the availability and access of appropriate personal protective equipment (PPE) for high-risk AGPs such as intubation. Lessons from previous experiences (severe acute respiratory syndrome [SARS]) reveal that a significant proportion of infections is related to breaches in the donning and doffing process.^6^ While every institution should have access to PPE for providers performing an AGP, it is important to ask the question of whether these recommendations are what is best for a provider in a room (negative pressure or not) preparing to intubate the sickest of COVID-19 patients. The question, therefore, beyond safe PPE is how does this PPE affect your ability to perform the stressful procedure? Does it restrict your peripheral vision, and will your face protection fog from your own tachypneic state or cause glare? Providers should liaise closely with their infection control experts regarding access to and training for donning and doffing PPE. Patients entering the room should be either “buddy checked” or signed off by an assigned PPE “supervisor” to ensure adequate donning and then again on leaving the room for the higher risk doffing procedure.

4.: **How should we approach support to preoxygenation?**

**Figure 2.**
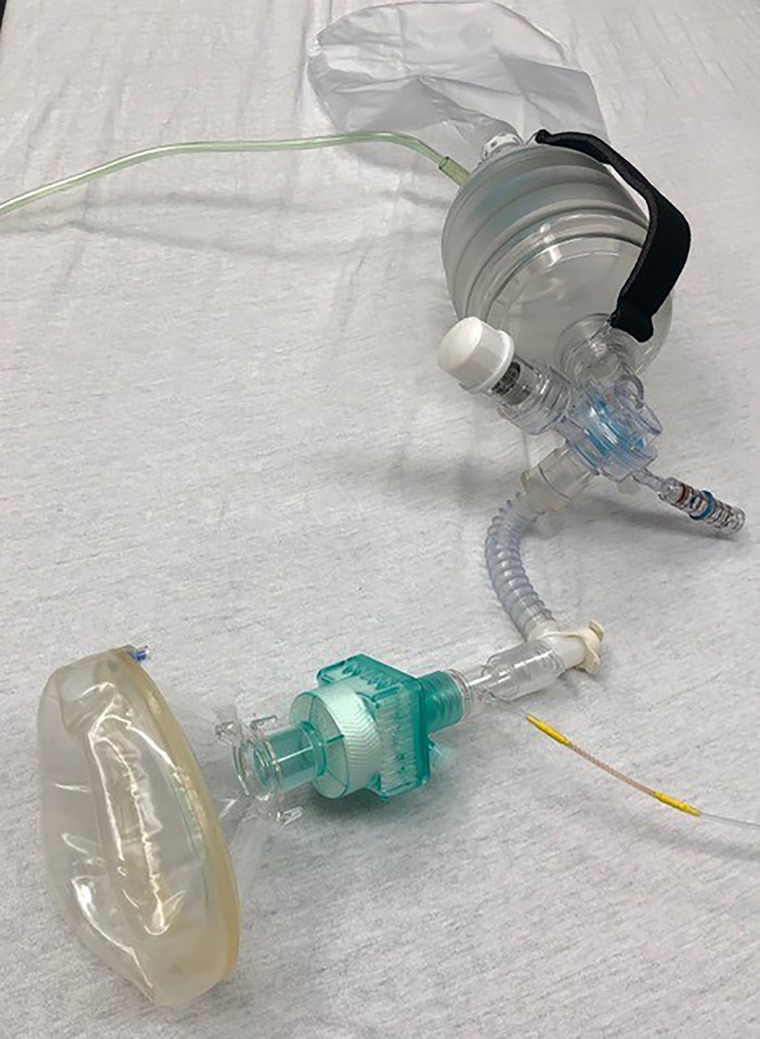
Modified BVM with Viral Filter, ETCO2, flexible mount (allows for more range of motion of device and less of a fulcrum at face) attached to BVM with PEEP valve and pressure monometer.

*Answer:* Preoxygenation in COVID-19 patients will deviate from familiar ED practice. *Disclaimer:* There is no concrete evidence to support specific no-risk preoxygenation techniques in this population. However, the overlying principle is to use the lowest flow necessary to achieve an acceptable saturation. Pushing flows to achieve higher oxygen saturation increases risk without benefit. What exactly does that mean? Aiming for an oxygen saturation of 90–92% may be reasonable, if achievable. It may initially mean having low flow (< 6 L/min) nasal prongs and then escalating to 15 L/min using a non-rebreather face mask), which is usually well tolerated. For most emergency physicians, preoxygenation will transition to using a bag-valve-mask (BVM) that can be purposely modified for COVID-19 patients (Figure 2; see also video: https://vimeo.com/406929923). The key difference from our standard equipment use is that from here on, anything applied to the face or trachea (mask or tube) needs a viral filter (Figure 2). Applying a tight-fitting mask before you are ready may create an uncooperative patient. The following sequence will create an aerosolization risk, which is why we are in full PPE for an AGP. Having a dissociative dose of ketamine ready to give slowly (delayed sequence intubation^7^) is critical. Do not squeeze the bag! When ready, you can place it directly over the patient's mouth or over the nasal prongs. Placing a mask over nasal prongs does create small risk of a leak that must be balanced against an uncooperative patient who will likely need the additional flow to generate positive end-expiratory pressure (PEEP). Remember, these patients have underlying shunt physiology (pneumonia, evolving acute respiratory distress syndrome) and so they are apnea-intolerant, meaning that, following RSI drug administration, these patients will further desaturate *very rapidly.*

5.: **What is the best approach to intubation in COVID-19 patients?**

*Answer:* Perform RSI and use a video laryngoscope (VL) as part of an old-fashioned “double setup.” (Be prepared to perform a cricothyrotomy.) Pretty simple here, awake intubations are essentially *contraindicated*. Period. Ideally using a video laryngoscope that keeps your face safely away from the patient's face. However, RSI alone is not an approach. Practice with and use checklists/visual aids which are accessible and ideally posted in the room (see Figure 1 and supplemental material). As part of your pre-brief, communicate the plan including your primary and alternative approach to intubation, what to do between intubation attempts, and what your exit and emergency strategy is (Plans ABCD, Figure 1). No airway carts should be in the room. Organize pre-packs with appropriately sized equipment for that patient (see supplemental material). Use a checklist that you and your team have practiced with and works for your environment (e.g., see supplemental material). Draw up your labelled medications for your RSI, rescue push-dose pressors, and begin a norepinephrine infusion at a starting dose based on hemodynamics. Have your bolus and infusion ready for post-intubation analgesia and sedation to take in the room. Keep it simple for your RSI. Use ketamine at 1.5 mg/kg and either high-dose succinylcholine or rocuronium at 1.5 mg/kg. Lower your ketamine dose if the shock index is > 1 (it is difficult to calculate to decimal points when your heart rate is elevated!). The choice of paralytic cannot influence success. *You can't get more paralyzed than paralyzed.* Give your drugs, WAIT (or risk cough and regurgitation), and go in on a “profoundly” paralyzed patient. Driver et al. achieved an FPS rate of 98% with routine use of a bougie in combination with a Macintosh blade VL device.^8^

An out-of-package bougie is straight with a coude tip and is meant for Macintosh blade devices. Recognize for some Macintosh VL devices that a slight bend on the distal portion of the bougie may be necessary. The nuances of VL use are beyond the scope of this article; however, use of a hyper-angulated VL can be a primary approach for those trained and confident with the nuances of tube delivery and/or be considered if an “optimized” Macintosh VL approach fails (see aimeairway.ca for procedure videos for laryngoscopy tips).

6.: **What if I fail to intubate?**

*Answer:* Breathe. Slow down. Yes, slow down. Place an oral airway, and apply oxygen via your BVM with two hands using a V-E grip jaw thrust with 10–15 cm of PEEP over nasal prongs at 5 L/min and your BVM at 15 L/min (apneic continuous positive airway pressure [apneic CPAP]; see Apneic CPAP https://vimeo.com/400368564). Don't look for the oxygen saturations to rise, but do ask for help if a second provider is available in PPE. You won't see an end-tidal CO2 trace unless you gently provide pressure support. Anytime you squeeze the bag, there is some risk to aerosolization; however, your patient has been rendered apneic. The risk of controlled ventilation (6–10 breaths over 1 minute) must be balanced against worsening hypoxemia that results in cardiac arrest (bad). A third option is your rescue supraglottic device (e.g., EMS i-gel®). If you are able to maintain saturations, you have to consider whether a second attempt at VL will be of value by you or your help. Alternatively, move to your exit strategy (see Figure 1). If you can't maintain oxygenation by either apneic CPAP, controlled ventilation, or a supraglottic device, employ your “emergency” double setup strategy and perform a cricothyrotomy.^9^^,^^10^

**KEY POINTS**

• Airway management of COVID-19 patients requires a paradigm shift from a focus primarily on patient-oriented outcomes to one that focuses on provider safety.
• RSI using a familiar VL device is the default method to secure the airway.
• Slow down to ensure patient and provider safety.
• Train in donning and doffing PPE, best practice airway skills wearing PPE, and as a team executing your plans.

## Supplementary material

For supplementary material accompanying this paper visit http://dx.doi.org/10.1017/cem.2020.353. click here to view supplementary material For updated COVID-19 airway management video and other support material, go to aimeairway.ca.

## References

[1] Murthy S, Gomersall CD, Fowler RA. Care for critically ill patients with COVID-19. JAMA 2020;epub, doi:10.1001/jama.2020.3633. 32159735

[2] Luo M, Cao S, Wei L, Precautions for intubating patients with COVID-19. Anesthesiology 2020;1. 10.1097/ALN.0000000000003288 PMC7155910 32195703

[3] Chen N, Zhou M, Dong X, Epidemiological and clinical characteristics of 99 cases of 2019 novel coronavirus pneumonia in Wuhan, China: a descriptive study. Lancet 2020;395(10223):507–13. 32007143 10.1016/S0140-6736(20)30211-7 PMC7135076

[4] Kovacs G, Law JA, Witter T. Airway Management Guidelines for Patients with Known or Suspected COVID-19 Infection. Nova Scotia Health Authority; 2020. Available at: http://policy.nshealth.ca/Site_Published/covid19/document_render.aspx?documentRender.IdType=6&documentRender.GenericField=&documentRender.Id=76497 (accessed April 9, 2020).

[5] Wax RS, Christian MD. Practical recommendations for critical care and anesthesiology teams caring for novel coronavirus (2019-nCoV) patients. Can J Anaesth 2020;epub, doi:10.1007/s12630-020-01591-x. 10.1007/s12630-020-01591-x PMC7091420 32052373

[6] Caputo KM, Byrick R, Chapman MG, Orser BJ, Orser BA. Intubation of SARS patients: infection and perspectives of healthcare workers. Can J Anaesth 2006;53(2):122–9. 16434750 10.1007/BF03021815

[7] Weingart SD, Trueger NS, Wong N, Delayed sequence intubation: a prospective observational study. Ann Emerg Med 2015;65(4):349–55. 25447559 10.1016/j.annemergmed.2014.09.025

[8] Driver BE, Prekker ME, Klein LR, Effect of use of a bougie vs endotracheal tube and stylet on first-attempt intubation success among patients with difficult airways undergoing emergency intubation. JAMA 2018;319(21):2179. 29800096 10.1001/jama.2018.6496 PMC6134434

[9] Brewster DJ, Chrimes NC, Do TBT, Consensus statement: Safe Airway Society principles of airway management and tracheal intubation specific to the COVID-19 adult patient group. Med J Aust 2020; epub, 1–36. Available at: https://www.mja.com.au/journal/2020/consensus-statement-safe-airway-society-principles-airway-management-and-tracheal (accessed March 31, 2020). 10.5694/mja2.50598 PMC7267410 32356900

[10] Royal College of Anaesthetists. COVID-19 airway management principles; 2020 Available at: https://icmanaesthesiacovid-19.org/covid-19-airway-management-principles (accessed March 31, 2020).

