# Supplementary material for: Just the Facts: Airway management during the coronavirus disease 2019 (COVID-19) pandemic
Source: CJEM. 2020 Mar 30:1–5. doi: 10.1017/cem.2020.353 (PMC7203167; doi:10.1017/cem.2020.353)
Supplement: Supplementary file 1 [file S148180352000353Xsup001.zip › CJEM/CoVID-19_Airway_Checklist-v10.pdf]

COVID-19 AIRWAY CHECKLIST

STOP BEFORE ENTERING ROOM

SLOW DOWN CHECK PPE

TEAM

- ☐ PPE Spotter
- ☐ Primary team
  - ☐ MD: Intubator/Lead
  - ☐ Airway assistant*
  - ☐ Clinical assistant*
- ☐ Support team
  - ☐ MD: Intubator assist + cardiac arrest lead
  - ☐ Airway assistant*
- ☐ Review roles

*RT, Medic, Nurse

EQUIPMENT

- ☐ AIRWAY ASSIST KIT
- ☐ INTUBTION KIT
- ☐ Supplies from COVID CART
- ☐ Two bougies
- ☐ Towels/ramp for positioning
- ☐ KIT DUMP Sheets
- ☐ Video-laryngoscopy check
- ☐ Ventilator + extra filter
- ☐ IV equipment
- ☐ Medications (w/flush):
  - RSI (Induction/paralytic)
  - Pre-dosed vasopressor
  - Post ETI sedation-analgesia

PLAN

- ☐ SUMMARIZE PLAN
  - ☐ Preoxygenation
  - ☐ Plan A: MAC VL + BOUGIE
  - ☐ Plan B: Alternative
  - ☐ Plan C: Exit strategy
  - ☐ Plan D: Emergency strategy
- ☐ Encountered difficulty
- ☐ Cardiac arrest
- ☐ Circuit disconnection

ADDRESS QUESTIONS ?

INTUBATION

PREOX

3-5min / SATS >90%

- ☐ Minimize flow
- ☐ NP<5LPM

+

- ☐ Filtered NRB <15LPM

OR

- ☐ Filtered BVM up to 15 lpm w/PEEP 10-15 cmH20

+

- ☐ NP<5 lpm

2hands

- ☐ Ketamine 1mg/kg PRN if dissociation needed

RSI/DSI

MAY NOT OBTAIN

- ☐ Head up 25°/Sniff position
- ☐ Ketamine 1-1.5mg/kg (if not given in preox OR no longer dissociated)
- ☐ Rocuronium 1.5 mg/kg
- ☐ Maintain airway
- ☐ 50 second count
- ☐ Place tube (see algorithm)

ANTICIPATE DE SAT

Closed loop Communication

POST INTUBATION

- ☐ Inflate cuff before ventilation
- ☐ Connect directly to ventilator w/viral filter
- ☐ Confirm tube placement by capnographic waveform - secure
- ☐ **Suction Required:** Clamp tube; connect inline suction
- ☐ **Hypotension:** Rescue Pressor + infusion PRN
  Norepi, Epi, Phenylephrine
- ☐ **Initial Ventilator:** Vt up to 8mL/kg if Driving pressure < 15, Pplat <30
  PEEP 10-12, FiO2 1.0, RR 16
  Reassess and titrate as needed
- ☐ **Sedation-Analgesia:** Ketamine/Propofol/Fentanyl
  Bolus doses should be available

CIRCUIT DISCONNECTION
Place finger over tube and attach filter

Exit one-by-one
Doff with a spotter

COVID-19 Adult Emergency Rapid Sequence Intubation Approach

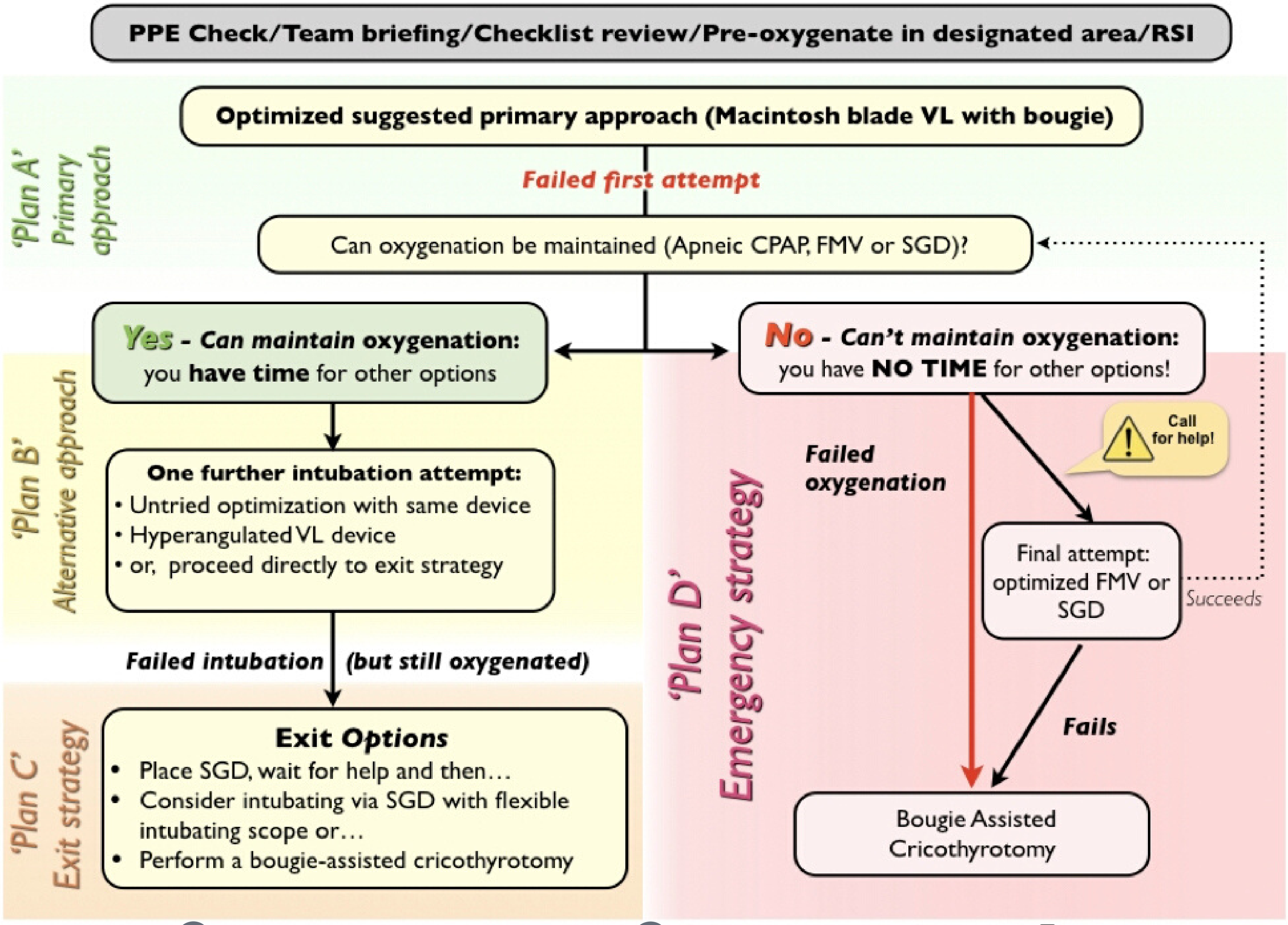

INTUBATION KIT

Regular ETT 8.0
Evac TT 7.5
VL stylet (1)
10cc syringe (2)
Lubricant (2)
Skin marker
#10 blade
Regular 6.0 TT

*add from cart:
Igel - sized
VL Mac blade sized
VL HA blade sized
Rigid Suction
Colorimetric capnography

AIRWAY ASSIST KIT

BVM with:
- PEEP
- Monometer
- Flexmount
- WFCO2
- Viral Filter
- Mask

OPA (red)
Tube secure device
10cc syringe (2)
NRB+Nasal prong
Extra viral filter
Inline suction
*Add tube clamp from cart

COVID CART (OUTISDE ROOM)

*not in kits

VL-Mac blade sized
VL-HA blade sized
Bougies
igel / SGA sized
Colorimetric Capnography
Tube clamps
Extra supplies
- NRB
- Nasal prong
- OPA
- Alt. Mask Sizes

Be Safe, Slow Down, Take care of yourself in these difficult times
