# Supplementary figures and images for: Just the Facts: Airway management during the coronavirus disease 2019 (COVID-19) pandemic
Source: CJEM. 2020 Mar 30:1–5. doi: 10.1017/cem.2020.353 (PMC7203167; doi:10.1017/cem.2020.353)

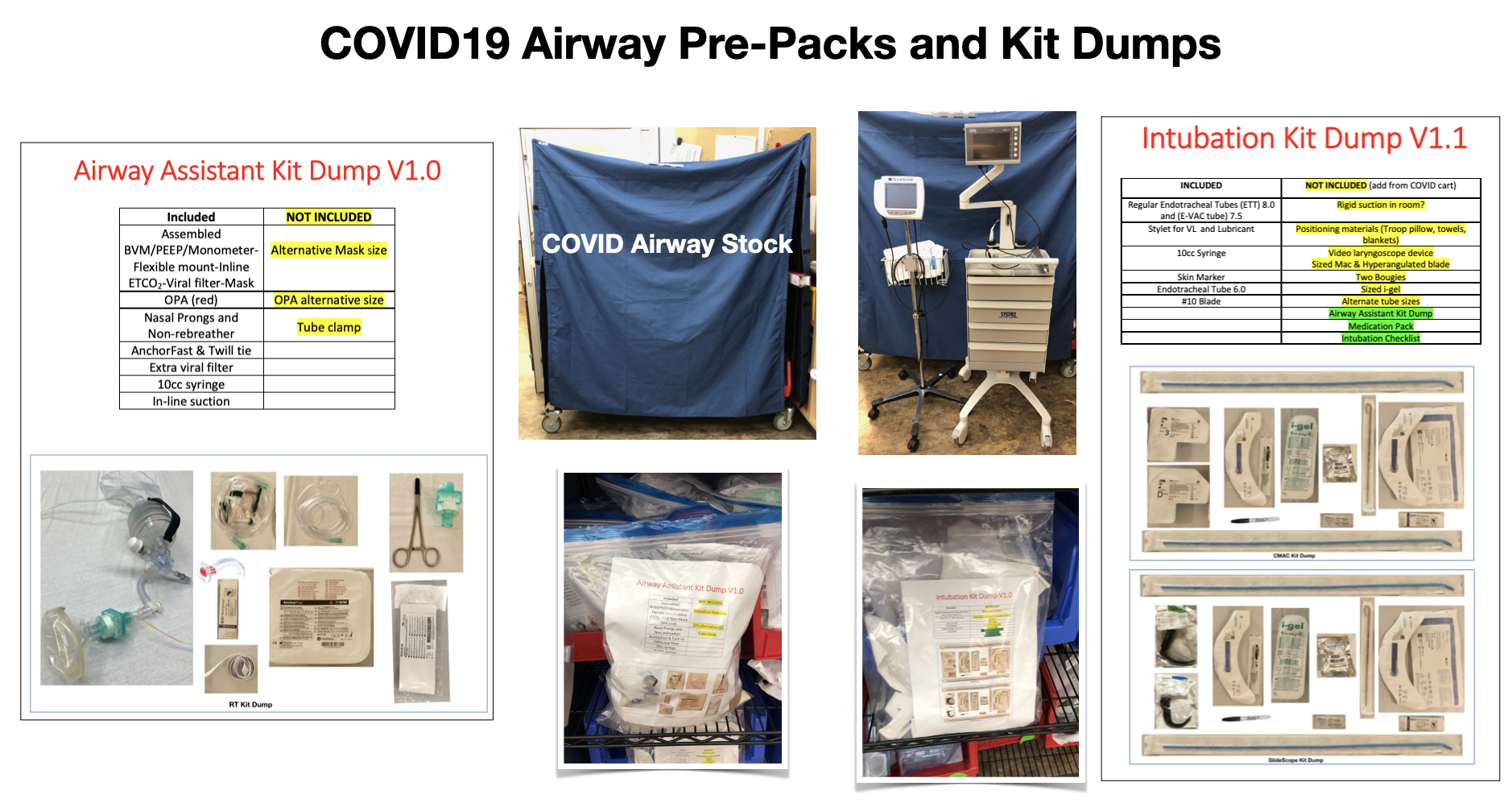

Supplement: Supplementary file 1 [file S148180352000353Xsup001.zip › CJEM/Covid19_Dump_kits.jpg]
